# Gut Microbiome Alterations Precede Cerebral Amyloidosis and Microglial Pathology in a Mouse Model of Alzheimer's Disease

**DOI:** 10.1155/2020/8456596

**Published:** 2020-05-24

**Authors:** Yijing Chen, Lihua Fang, Shuo Chen, Haokui Zhou, Yingying Fan, Li Lin, Jing Li, Jinying Xu, Yuewen Chen, Yingfei Ma, Yu Chen

**Affiliations:** ^1^The Brain Cognition and Brain Disease Institute, Shenzhen Institutes of Advanced Technology, Chinese Academy of Sciences, Shenzhen-Hong Kong Institute of Brain Science-Shenzhen Fundamental Research Institutions, Shenzhen, Guangdong, China; ^2^Institute of Synthetic Biology, Shenzhen Institutes of Advanced Technology, Chinese Academy of Sciences, Shenzhen, Guangdong, China; ^3^University of Chinese Academy of Sciences, Beijing, China; ^4^Guangdong Provincial Key Laboratory of Brain Science, Disease and Drug Development, HKUST Shenzhen Research Institute, Shenzhen-Hong Kong Institute of Brain Science-Shenzhen Fundamental Research Institutions, Shenzhen, Guangdong, China

## Abstract

Emerging evidence suggests that the gut microbiome actively regulates cognitive functions and that gut microbiome imbalance is associated with Alzheimer's disease (AD), the most prevalent neurodegenerative disorder. However, the changes in gut microbiome composition in AD and their association with disease pathology, especially in the early stages, are unclear. Here, we compared the profiles of gut microbiota between APP/PS1 transgenic mice (an AD mouse model) and their wild-type littermates at different ages by amplicon-based sequencing of 16S ribosomal RNA genes. Microbiota composition started diverging between the APP/PS1 and wild-type mice at young ages (i.e., 1–3 months), before obvious amyloid deposition and plaque-localized microglial activation in the cerebral cortex in APP/PS1 mice. At later ages (i.e., 6 and 9 months), there were distinct changes in the abundance of inflammation-related bacterial taxa including *Escherichia*-*Shigella*, *Desulfovibrio*, *Akkermansia*, and *Blautia* in APP/PS1 mice. These findings suggest that gut microbiota alterations precede the development of key pathological features of AD, including amyloidosis and plaque-localized neuroinflammation. Thus, the investigation of gut microbiota might provide new avenues for developing diagnostic biomarkers and therapeutic targets for AD.

## 1. Introduction

Alzheimer's disease (AD) is a neurodegenerative disease and form of dementia that severely impairs cognitive functions and daily activities. In AD, the major pathological changes in the brain are elevated levels of extracellular amyloid plaques and intracellular neurofibrillary tangles [1]. The combinatorial effects of genetic and environmental factors are thought to contribute to the disease pathogenesis. Moreover, recent accumulating evidence suggests that gut microbiota dysbiosis and microbial infection might be associated with AD etiology [2–4].

The complex gut microbiota in the mammalian gastrointestinal ecosystem participates in a plethora of physiological processes [5, 6]. Pathological changes in the gut microbiome not only lead to gut dysfunction but also are associated with central nervous system (CNS) disorders such as neurodegeneration, autism, and depression [7–11]. Gut microbiota can affect CNS functions through multiple ways, for example, by releasing neurotransmitters (e.g., acetylcholine, GABA, dopamine, and serotonin) and endotoxins that gain access to the brain via blood circulation, triggering the secretion of proinflammatory cytokines (e.g., IL-1*β*, IL-6, and IL-17A) or anti-inflammatory cytokines (e.g., IL-4 and IL-10) by mucosal immune cells that regulate immune activity in the CNS, as well as activating the gut vagus nerve, which influences multiple brain regions involving the limbic cortex, hippocampus, amygdala, striatum, and hypothalamus [12–15]. In turn, the CNS might influence gut microbiota composition through the reciprocal gut-brain axis: stress and negative emotions such as anxiety and depression might cause the release of neurotransmitters (including noradrenaline, adrenaline, and corticosterone) through the hypothalamic-pituitary-adrenal axis and sympathetic nervous system, which could influence gut physiology and alter microbiota composition [16, 17]. In contrast, positive emotions such as happiness and positive affect are associated with lower expression of inflammatory genes in the immune cells and lower levels of proinflammatory cytokines such as IL-6 and TNF-*α* [18–20].

Emerging evidence suggests that microbial infection is a key factor in the etiopathogenesis of AD, opening new avenues for therapeutic development [21]. Indeed, recent studies revealed that gut microbiota composition and diversity are altered in AD patients and animal models [2, 22–27]. Moreover, pathogenic microbes can induce amyloid-beta (A*β*) oligomerization and promote chronic inflammatory responses that contribute to AD progression [4, 28, 29]. However, there is a lack of evidence that the microbiota is altered in early AD or in the long term. Therefore, the changes in the microbiota profiles in AD require further investigation.

In the present study, we investigated longitudinal changes in the gut microbiome in an AD mouse model starting from an early age. We found that compared to wild-type littermates, the microbiota composition in AD mice began to diverge significantly from an early age (before the detection of A*β* plaques in the cortex) and subsequently diverged even further. Thus, our findings suggest that gut microbiota composition may be associated with the progression of AD pathology.

## 2. Materials and Methods

### 2.1. Animals and Sample Collection

We obtained APP/PS1 double-transgenic mice (B6C3-Tg(APPswe, PSEN1dE9)85Dbo/J; stock number 2010-0001), a mouse model of AD in a C57BL/6 background, from the Nanjing Biomedical Research Institute of Nanjing University. We housed APP/PS1 mice and their age-matched wild-type (WT) littermates together (*n* = 4 mice/cage) under specific pathogen-free conditions and at a constant temperature (24°C) in a 12 h light/dark cycle, with autoclaved water and standard chow *ad libitum*. We collected fresh fecal samples from individual male mice at 1, 2, 3, 6, and 9 months of age (*n* = 14–24 for the 1-, 2-, 3-, and 9-month-old groups, *n* = 31–34 for the 6-month-old group; Table 1), froze them immediately, and stored them at −80°C before analysis. This study was performed in accordance with the recommendations of the National Care and Use of Animals Guidelines (China) and approved by the Institutional Animal Care and Use Committee (IACUC) of the Shenzhen Institutes of Advanced Technology, Chinese Academy of Sciences.

### 2.2. Fecal DNA Extraction and Sequencing

We extracted fecal DNA from mouse feces using the DNeasy PowerSoil Kit (QIAGEN, USA) according to the manufacturer's instructions. We measured the concentration and quality of the extracted DNA by NanoDrop 2000 (NanoDrop Technologies, Thermo Scientific, USA) and 1.2% agarose gel electrophoresis. We subsequently amplified 16S ribosomal RNA (16S rRNA) genes with a set of primers that target the V3-V4 hypervariable regions of the bacterial 16S rDNA (see Supplementary Table S1 for detailed information). We performed polymerase chain reaction (PCR) with PCR mix (Transgene, China), fecal DNA template, primers, and sterilized ultrapure water in a total volume of 50 *μ*L according to the manufacturer's instructions. The cycling conditions were as follows: initial denaturation at 95°C for 5 minutes; 35 cycles of denaturation at 95°C for 30 seconds, annealing at 60°C for 30 seconds, and extension at 72°C for 45 seconds; and final extension at 72°C for 10 minutes. We purified PCR amplification products using the QIAquick PCR Purification Kit (QIAGEN, USA). We used the purified PCR products for library preparation and high-throughput sequencing on an Illumina HiSeq 1500 sequencer (Novogene Bioinformatics Technology, China).

### 2.3. Sequencing Data and Statistical Analyses

To analyze the high-throughput sequencing data, we performed comprehensive bioinformatic and statistical analyses as previously described [30, 31]. First, we used FLASH v1.2.7 to assemble the sequence reads via overlap sequences of the paired-end reads after high-throughput sequencing. Then, we obtained high-quality reads after removing low-quality reads (Phred quality score < Q30), ambiguous bases, and adapter sequences. We used Trimmomatic v0.33 to trim adapters and UCHIME v4.2 to remove chimeric sequences [32]. We classified the remaining sequences into operational taxonomic units (OTUs) according to their identities. Sequences with >97% identity were clustered into an OTU, and the relative abundances of OTUs were estimated. We used QIIME and UCLUST to select the most abundant sequence as a representative sequence for each OTU [33]. We classified each OTU according to the SILVA 16S rRNA gene reference alignment database and assigned each a taxonomic identity. We classified the taxon abundance of each sample according to phylum, class, order, family, and genus by using the RDP and GreenGene databases. We calculated UniFrac distance using phylogenetic information, which was based on the phylogeny inference of sequence alignment determined by PyNAST.

We determined alpha diversity from rarefied OTU tables by using mothur software, which included the Chao1, ACE, Shannon, Simpson, observed species (i.e., OTU number), and coverage indexes [34]. Meanwhile, to compare diversity between groups, we calculated beta diversity by using mothur software, namely, principal coordinate analysis (PCoA), based on unweighted and weighted UniFrac phylogenetic distance [35]. We statistically analyzed alpha diversity by using the Wilcoxon rank-sum test and beta diversity by using permutational multivariate analysis of variance (PERMANOVA) in the *vegan* package in R [30]. We performed the Wilcoxon rank-sum test to detect the significance of the relative abundance of gut microbiota between sample groups at the phylum, family, and genus levels; the results were corrected by false discovery rate (FDR). We used linear discriminant analysis effect size analysis to determine the bacterial taxa that differed significantly between groups [36]. The level of significance was set at *p* < 0.05.

### 2.4. Immunohistochemistry

On the day of sacrifice, we weighed and deeply anaesthetized mice by intraperitoneal injection of 0.6% pentobarbital (70 mg/kg) before transcardially perfusing them with 4% paraformaldehyde (PFA) in phosphate-buffered saline (PBS, pH 7.4). Then, we excised the brains and postfixed them overnight in 4% PFA before transferring them to 30% sucrose solution for cryoprotective procedures. We performed immunostaining on free-floating 20 *μ*m cryostat sections. We blocked sections in 10% goat serum in TBS-T (0.1% Triton X-100, Sigma) for 1 h before incubation with anti-A*β*_17–24_ (4G8, 1 : 1,000, Covance) and anti-Iba1 (1 : 500, 019-19741, Wako) primary antibodies overnight at 4°C. After washing with TBS-T, we incubated the sections with anti-mouse-Alexa 488- and anti-rabbit-Alexa 568-conjugated secondary antibodies (1 : 1,000, Invitrogen) for 2 h at room temperature while protecting them from light. We counterstained the sections with DAPI (4′, 6-diamidino-2-phenylindole) and mounted them with Fluoromount-G (SouthernBiotech). We acquired images at 10x, 20x, and 63x magnification using a Zeiss LSM 880 confocal microscope.

### 2.5. Quantification of A*β* Plaque Burden and Iba1^+^ Microglia

We quantified A*β* plaque staining by using the *Analyze Particles* tool in ImageJ software. For each section, we set intensity thresholds to eliminate background fluorescence and quantified 4 sections for each mouse (*n* = 4 mice for each age group) throughout the study. The A*β* plaque load percentage was determined by dividing the total plaque area by the area of the cerebral cortical region of each section [37]. The percentage of Iba1^+^ microglial immunoreactivity was calculated using a similar method (i.e., by using the *Analyze Particles* tool in ImageJ to measure the total Iba1^+^ area and dividing it by the area of the cerebral cortex of each section) [38]. Plaque-localized microglial cell numbers in APP/PS1 mice were counted using ImageJ software and compared to those in an arbitrary location in the cortex of WT mice. Specifically, Iba1-positive somas colocalized with DAPI and within the 200 *μ*m vicinity of A*β* plaques were identified as plaque-localized microglia [27]. The number of activated microglia with deramified morphology around the plaque was quantified manually in each section [39]. Statistical analysis was performed by using GraphPad Prism software. To compare the A*β* plaque load among the 5 age groups (1, 2, 3, 6, and 9 months) in APP/PS1 mice, we performed one-way ANOVA followed by the Bonferroni multiple-comparison *post hoc* analysis. For the comparisons of Iba1^+^ microglia between the WT and APP/PS1 mice at different time points, we applied two-way ANOVA followed by the Bonferroni multiple-comparison test. A *p* value < 0.05 was considered statistically significant.

## 3. Results

### 3.1. Characterization of Gut Microbiota Sequencing Data

To investigate how intestinal microbiota is regulated during AD development, we systematically examined age-related changes in the microbiota composition in APP/PS1 mice in comparison to age-matched WT littermates (Table 1). There was a total of 9,987,522 V3-V4 16S rRNA sequence reads from 203 samples, with an average of 47,738 sequence reads per sample. The average length of sequence reads was 315 bp (Supplementary Figure S1). Sequences were clustered into OTUs according to sequence identity.

### 3.2. Alterations in Gut Microbiome Composition in APP/PS1 Mice

**Figure 1:**
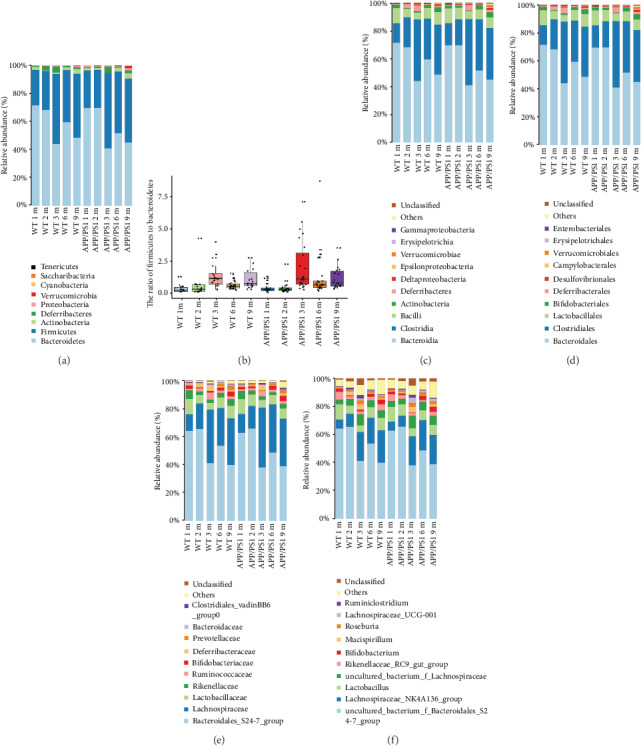
Changes in microbe composition in mouse feces along development and aging. (a) The relative abundance of top microbes in mouse feces is shown at the rank of phylum. (b) The ratio of the abundance of Firmicutes to Bacteroidetes in all groups. (c–f) Profiles of the microbes in mouse feces at the rank of (c) class, (d) order, (e) family, and (f) genus. 1 m, 2 m, 3 m, 6 m, and 9 m indicate mice at 1, 2, 3, 6, and 9 months of age, respectively.

**Figure 2:**
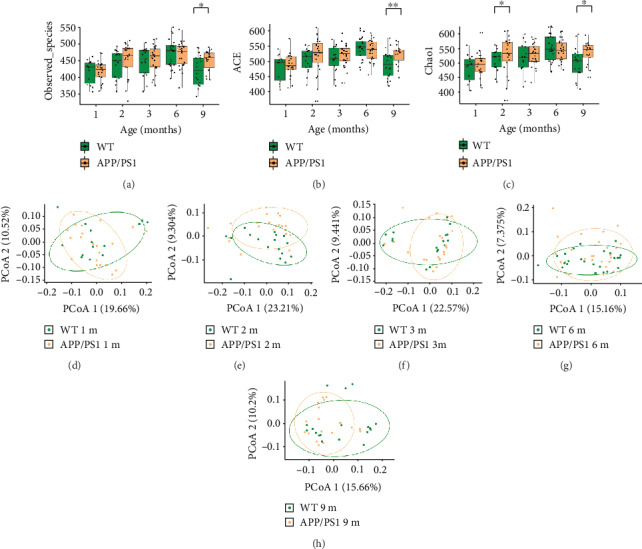
Age-dependent alteration of gut microbiome composition in Alzheimer's disease mice. (a–c) Alpha diversity was determined on the basis of the numbers of observed species as well as the ACE and Chao1 indexes. (d–h) Principal coordinate analysis (PCoA) based on unweighted and weighted UniFrac phylogenetic distance was compared between the wild-type (WT) and APP/PS1 mice at different ages. ^∗^*p* < 0.05, ^∗∗^*p* < 0.01. 1 m, 2 m, 3 m, 6 m, and 9 m indicate mice at 1, 2, 3, 6, and 9 months of age, respectively.

To delineate the changes in gut microbiota with age, we compared microbial abundance at the phylum, class, order, family, and genus levels between the WT and APP/PS1 mouse feces. Across all age groups in both the WT and APP/PS1 mice, the dominant phyla were Firmicutes and Bacteroidetes (Figure 1(a)). The ratio of the abundance of Firmicutes to Bacteroidetes shifted with age; the changes were similar between the WT and APP/PS1 mice (Figure 1(b), Table 2). We observed similar trends in the top 2 bacterial taxa at the rank of class, order, family, and genus (Figures 1(c)–1(f)). To further characterize gut microbiota composition, we performed alpha diversity analysis, which measures the abundance and richness of OTUs within a given sample. In both the WT and APP/PS1 mice, the OTU number, ACE index, and Chao1 index trended towards increased diversity and richness from 1 to 6 months and a decline in diversity at 9 months (Figures 2(a)–2(c)). However, compared to age-matched WT mice, the microbiota of 9-month-old APP/PS1 mice exhibited greater richness and diversity with respect to the OTU number, ACE index, and Chao1 index (Wilcoxon rank-sum test; OTU number: *p* = 0.021; ACE: *p* = 0.008; Chao1: *p* = 0.012). Surprisingly, at 2 months, APP/PS1 mice also exhibited greater richness with respect to the Chao1 index than WT mice (Wilcoxon rank-sum test; Chao1: *p* = 0.045) (Table 3). There were no significant differences in the Simpson, Shannon, or coverage indexes between the WT and APP/PS1 mice at any age (data not shown).

Meanwhile, analysis of beta diversity using PCoA and unweighted UniFrac demonstrated significant differences in microbiome composition between the WT and APP/PS1 mice at 2 and 6 months (PERMANOVA; WT vs. APP/PS1 at 2 months: *p* = 0.035; WT vs. APP/PS1 at 6 months: *p* = 0.045) (Figures 2(d)–2(h), Table 4). These results reveal that the composition and community structures of the gut microbiota are altered in APP/PS1 mice starting from an early age. To provide a more intuitive representation of the differences in the distribution of OTU abundance between the WT and APP/PS1 mice at each age, we plotted cluster heatmaps of differentially abundant taxa at the OTU level (Supplementary Figure S2). The differential abundance of OTUs revealed that compared to WT mice, APP/PS1 mice had significantly altered gut microbiome composition across all age groups.

### 3.3. Differentially Represented Bacterial Taxa in APP/PS1 Mice

**Figure 3:**
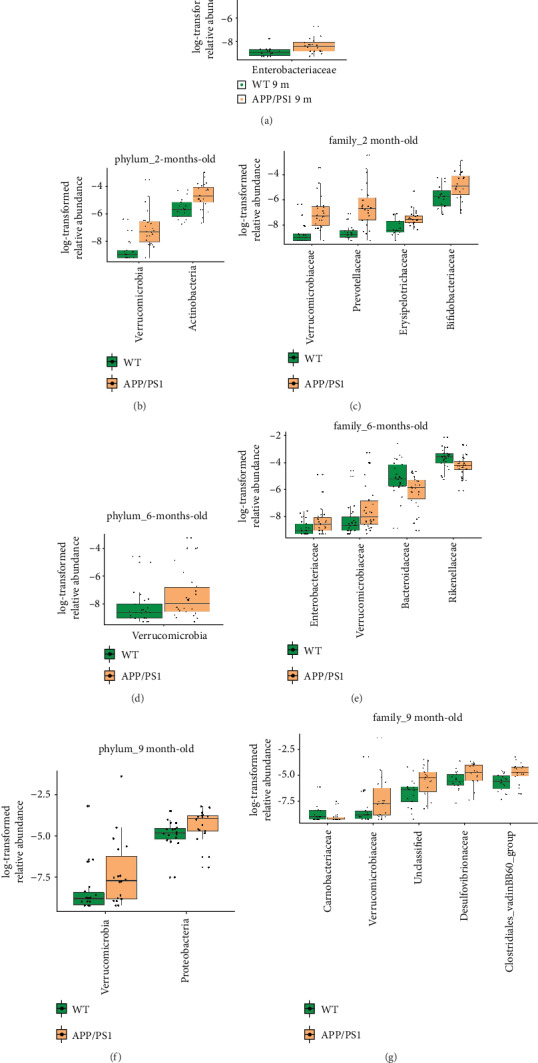
Variation of bacterial composition at the phylum and family levels in Alzheimer's disease mouse model feces. Data are presented as log-transformed relative abundance. (a–g) There were significant differences in the bacterial phyla and families with relative abundance ≥ 0.01% between the APP/PS1 and wild-type (WT) mice at 1, 2, 6, and 9 months of age, respectively (*p* < 0.05, false discovery rate- (FDR-) corrected).

**Figure 4:**
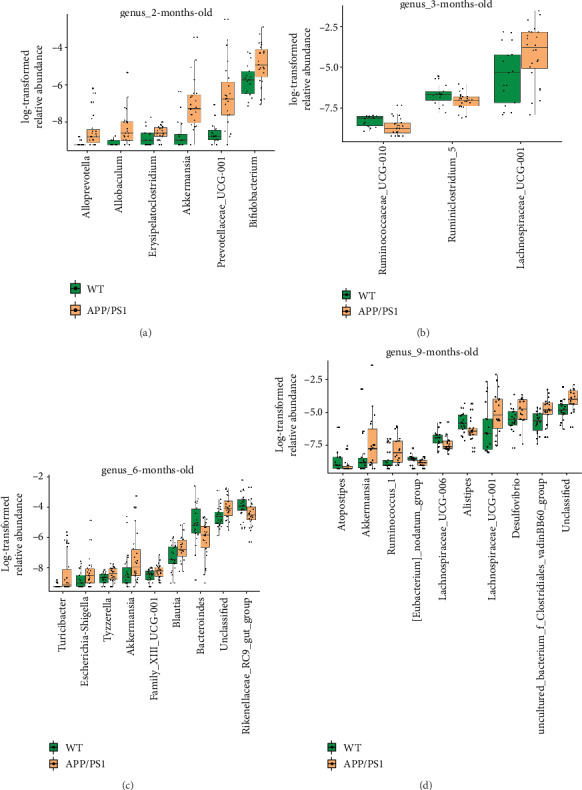
Variation of bacterial composition at the genus level in Alzheimer's disease mouse model feces. Data are presented as log-transformed relative abundance. (a–d) There were significant differences in the bacterial genera with relative abundance ≥ 0.01% between the APP/PS1 and wild-type (WT) mice at 2, 3, 6, and 9 months of age, respectively (*p* < 0.05, FDR-corrected).

Linear discriminant analysis effect size analysis showed that the gut microbial communities in both the WT and APP/PS1 mice changed dynamically with age, with most differentially abundant taxa being present in 9-month-old mice (*p* < 0.05) (Supplementary Figure S3). We subsequently applied multiple comparisons to analyze microbial taxa that were differentially represented between the WT and APP/PS1 mice at the phylum and family levels (Figure 3) and genus level Figure 4, summarized in Supplementary Table S2). Among the identified taxa, we focused on the taxa with occupancies ≥ 0.01% at each taxon level [25]. Compared to age-matched WT mice, 1-month-old APP/PS1 mice exhibited a significantly greater relative abundance of Enterobacteriaceae (phylum Proteobacteria) (Figure 3(a)). In addition, compared to age-matched WT mice, the relative abundance of phylum Verrucomicrobia and family Verrucomicrobiaceae was significantly higher in APP/PS1 mice at 2, 6, and 9 months, respectively (Figures 3(b)–3(g)). Moreover, the relative abundance of phyla Actinobacteria and Proteobacteria increased significantly in APP/PS1 mice at 2 and 9 months, respectively (Figures 3(b) and 3(f)). Besides Verrucomicrobiaceae, other bacterial families such as Prevotellaceae, Erysipelotrichaceae, and Bifidobacteriaceae were more abundant in APP/PS1 mice than in WT mice at 2 months (Figure 3(c)). There were no significant differences in microbiota composition between the 3-month-old APP/PS1 and WT mice at the phylum or family level (data not shown). Compared to age-matched WT controls, 6-month-old APP/PS1 mice exhibited significantly greater abundance of Enterobacteriaceae and Verrucomicrobiaceae as well as significantly lower abundance of Bacteroidaceae and Rikenellaceae (Figure 3(e)). By 9 months, the abundance of Verrucomicrobiaceae, Desulfovibrionaceae, Clostridiales_vadinBB60_group, and an unclassified taxon increased significantly in APP/PS1 mice, whereas that of Carnobacteriaceae decreased (Figure 3(g)).

At the genus level, compared to age-matched WT mice, APP/PS1 mice exhibited significantly greater abundance of *Akkermansia* (phylum Verrucomicrobia) at 2, 6, and 9 months, respectively (Figures 4(a), 4(c), and 4(d)). In addition, at 2 months, *Prevotellaceae_UCG*-*001*, *Bifidobacterium*, *Erysipelatoclostridium*, *Allobaculum*, and *Alloprevotella* were more abundant in APP/PS1 mice than WT mice (Figure 4(a)). There were no 5significant differences in microbiota composition at the genus level between the APP/PS1 and WT mice at 1 month (data not shown). At 3 months, compared to age-matched WT mice, *Lachnospiraceae_UCG_001*, a member of Lachnospiraceae in phylum Firmicutes, was more abundant in APP/PS1 mice, whereas the abundance of *Ruminococcaceae_UCG_010* and *Ruminiciostridium_5* (both phylum Firmicutes) was lower in APP/PS1 mice (Figure 4(b)). At 6 months, compared to WT mice, *Escherichia*-*Shigella*, *Akkermansia*, *Blautia*, *Turicibacter*, *Tyzzerella*, *Family_XIII_UCG*-*001*, and an unclassified taxon were more abundant in APP/PS1 mice, whereas *Bacteroides* and *Rikenellaceae_RC9_gut_group* were less abundant in APP/PS1 mice (Figure 4(c)). Finally, at 9 months, compared to WT mice, *Desulfovibrio*, *Akkermansia*, *uncultured_bacterium_f_Clostridiales_vadinBB60_group*, *Lachnospiraceae_UCG*-*001*, *Ruminococcus_1*, and an unclassified genus were more abundant in APP/PS1 mice, whereas *Atopostipes*, *Eubacterium_nodatum_group*, *Alistipes*, and *Lachnospiraceae_UCG*-*006* were less abundant in APP/PS1 mice (Figure 4(d)). These results collectively indicate that the abundance of several gut bacteria begins to differ significantly between the WT and APP/PS1 mice during the early stages of AD development and that these differences increase with age.

### 3.4. Association of Microglia with A*β* Plaques in the Cerebral Cortex in APP/PS1 Mice

**Figure 5:**
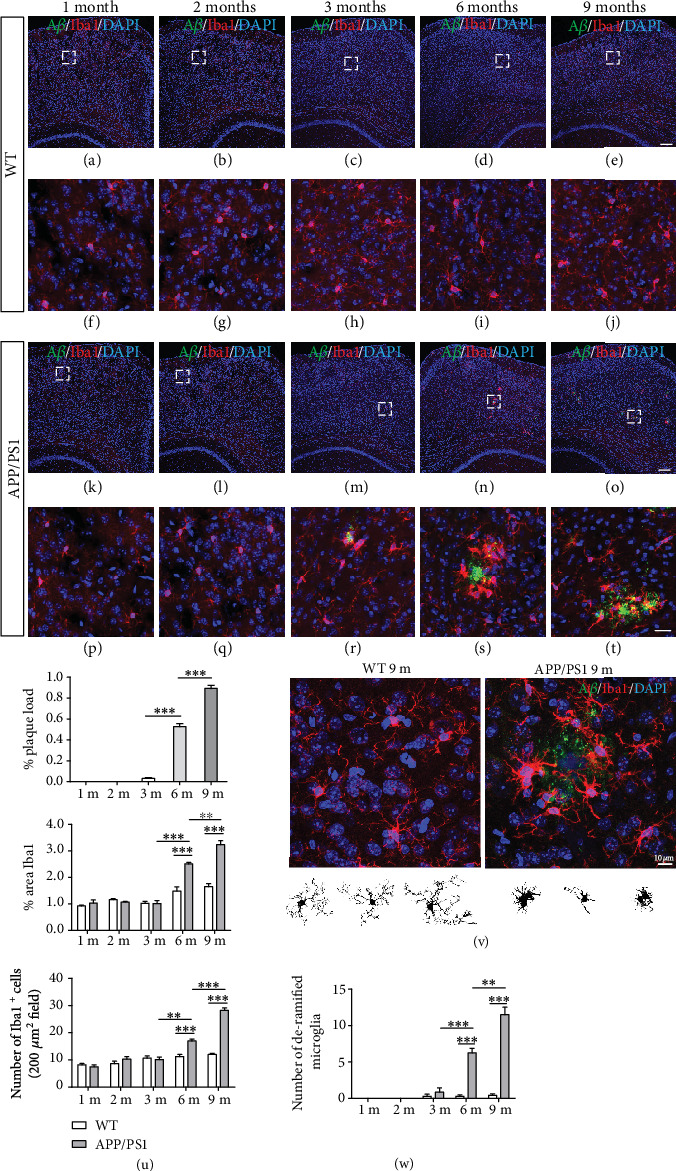
Increased amyloidosis and plaque-localized microglial activation in Alzheimer's disease mouse models during aging. Brain sections derived from the representative wild-type (WT) and APP/PS1 mice at 1, 2, 3, 6, and 9 months of age were immunolabeled with anti-Iba1 (red) and amyloid-beta (A*β*, green) antibodies and counterstained with DAPI. (a–e) and (k–o) are *z*-stack maximum projection images of the WT and APP/PS1 mice, respectively; (f–j) and (p–t) are higher-magnification images of the areas denoted by white rectangles, respectively. (u) Quantification of the amyloid-beta (A*β*) plaque load, Iba1 immunoreactivity, and plaque-localized Iba1^+^ microglia in APP/PS1 mice at 1, 2, 3, 6, and 9 months of age (*n* = 4 per age group). (v) Upper panels: representative images of plaque-localized Iba1^+^ microglial cells in the cortex in 9-month-old APP/PS1 mice; lower panels: representative images showing the morphology of microglial cells. (w) Quantification of deramified microglia in APP/PS1 mice at 1, 2, 3, 6, and 9 months of age versus their WT controls (*n* = 4 per age group). Data are presented as mean ± SEM. ^∗∗^*p* < 0.01, ^∗∗∗^*p* < 0.001. Scale bars: 100 *μ*m (a–e, k–o), 25 *μ*m (f–j, p–t), and 10 *μ*m (v).

To further examine the relationships between gut microbiota alterations and neuroinflammatory states in AD pathology, we analyzed the reactive gliosis surrounding A*β* plaques across all age groups. Given that Iba1 is commonly used as a general microglial marker in the brain, we performed the immunofluorescence staining with anti-Iba1 together with A*β* antibodies to reveal the distribution of microglia in relation to A*β* plaques in APP/PS1 mice. In 1- and 2-month-old APP/PS1 mice, there were no A*β* plaques in the cerebral cortex, and the microglia were evenly distributed (Figures 5(k) and 5(l); higher-magnification images in Figures 5(p) and 5(q), respectively). Very little A*β* staining was detected in the cortex in 3-month-old APP/PS1 mice, whereas obvious A*β* plaques were observed at 6 and 9 months, with the microglia clustered around them (Figures 5(m)–5(o); higher-magnification images in Figures 5(r)–5(t), respectively). On the other hand, there were no A*β* plaques in age-matched WT mice, and microglia were evenly distributed in the cortex in WT mice at all ages (Figures 5(a)–5(e); higher-magnification images in Figures 5(f)–5(j), respectively). Quantification of Iba1 immunoreactivity confirmed a significant increase in activated microglia and plaque-localized and deramified microglia in the cortex in APP/PS1 mice at 6 and 9 months (Figures 5(u)–5(w)). Given that we identified significant differences in microbial abundance between APP/PS1 mice and wild-type control mice at the early stages (i.e., 1–3 months) when A*β* plaque accumulation and microglial activation have not occurred in the cerebral cortex, our results suggest that alteration of the gut microbiome profile occurs prior to amyloidosis and plaque-localized microglial activation in the brain of AD model mice.

## 4. Discussion

Recent studies have unveiled a link between gut microbiota homeostasis and AD progression. However, the changes in microbiota profiles during AD development, especially in the early stages, are unclear. Therefore, in the present study, to examine the association between gut microbiota composition and the progression of AD pathology, we compared the fecal microbiome of WT and AD model mice (i.e., APP/PS1 mice) starting from an early age. We observed a shift in gut microbiota composition in the young adult WT and APP/PS1 mice as well as a tendency of microbial richness and diversity to decline in older mice. Our results are consistent with previous results showing that APP/PS1 mice exhibit greater richness in gut microbiota than WT mice by the Chao1 index, without a significant difference in the Simpson diversity between the 2 groups [24]. Microbial diversity usually increases after birth, and microbial composition changes gradually during late childhood, adolescence, and adulthood [40, 41]. Furthermore, reduced microbial diversity is associated with aging or poorer health conditions in humans [42–44]. The loss of diversity and decreased temporal compositional stability in elderly people are related to general health as well as to confounding factors such as diet and constrained lifestyles [45]. Recent studies showed that gut microbiota composition in AD patients differs from that in normal controls recruited from the same geographical area and shared dietary food habits; this indicates that the alteration of gut microbiota in AD patients may not be due to diet or comorbidities [23]. Our results consistently show a trend towards increased diversity and richness from 1 to 6 months and decreased diversity at 9 months in both the WT and APP/PS1 mice, indicating aging as a general factor influencing microbial composition. However, our results further demonstrate that the progression of AD is associated with additional changes in the abundance of inflammation-related gut microbiome composition against a background of age-dependent alteration of diversity. This finding indicates that the condition of AD may impose extra pressure on the regulation of microbial composition and abundance during aging.

We also identified several bacterial taxa whose abundance differed between the WT and APP/PS1 mice at different ages. Notably, compared to WT mice, significant differences in microbial abundance emerged at early ages in APP/PS1 mice (i.e., 1–3 months), while only obvious A*β* plaques and plaque-localized microglial activation were observed in the cerebral cortex in APP/PS1 mice older than 6 months. These findings indicate that the changes in microbial composition occur prior to the detection of obvious amyloidosis in the cerebral cortex in APP/PS1 mice. Moreover, we found that the differences in bacterial taxa between the WT and APP/PS1 mice increased with age, specifically at 6 and 9 months, which coincides with the progression of amyloid plaque formation and microglial activation in the APP/PS1 mouse brain. These findings suggest that gut microbial composition might be associated with AD pathogenesis and that such alterations might serve as potential diagnostic biomarkers and/or therapeutic targets for AD. There are some reports on gut microbiota composition in the APP/PS1 mouse model. However, as most of these studies focus on microbial changes in adult mice [25], there is a lack of evidence regarding whether and how the microbiota is altered in early AD. Previous longitudinal studies have used different time points and only revealed the changes of the gut microbiome at later stages without direct comparison to amyloidosis or microglial activation [24, 26]. Our results are consistent with previous data that the gut microbial composition in AD mice shifts towards an inflammation-related bacterial profile in the feces of APP/PS1 mice as compared to their age-matched WT controls [24–26]. We also observe higher abundance of *Escherichia*-*Shigella* in our study, which was identified in AD patients [2] but has not been reported in other studies with APP/PS1 mice.

The inflammatory-infectious hypothesis of AD has elucidated the essential role of gut microbiota in the initiation of systemic inflammation, which leads to neuroinflammatory responses in the brain [46]. The gut microbiota might regulate the development and maturation processes of microglia and astrocytes as well as peripheral immune responses; in turn, the activation of innate immune responses might modulate the homeostasis of intestinal bacterial communities [47–49]. For example, Proteobacteria are implicated in immunological and inflammatory reactions and act as a microbial signature of dysbiosis in disease states [50]. Accordingly, altered Proteobacteria composition has been found in patients with AD, irritable bowel syndrome, or Parkinson's disease [22, 51, 52]. Similarly, in the present study, the abundance of Enterobacteriaceae, a bacterial taxon belonging to Proteobacteria, was elevated in APP/PS1 mice as early as 1 month old. Meanwhile, elevated abundance of Enterobacteriaceae has been reported in the feces of children with autism and patients with Parkinson's disease, suggesting that these bacteria might be involved in CNS disorders [53–56]. Enterobacteriaceae includes many pathogenic bacteria such as *Escherichia coli* and *Shigella* spp., which can produce endotoxins that are released into the bloodstream and consequently induce systemic inflammatory responses [57–59]. Our results indicate that young APP/PS1 mice have an aberrant gut microbial composition at the age when A*β* deposits have not yet developed in the brain. Moreover, the abundance of Proteobacteria was elevated in APP/PS1 mice at later ages; specifically, we observed increases in *Escherichia*-*Shigella* at 6 months and *Desulfovibrio* at 9 months. Recent studies show that A*β* protects against microbial infection in the 5xFAD transgenic mouse model, in the nematode *Caenorhabditis elegans*, and in cultured cell models of AD [4, 60]. Concordantly, a cohort study found that compared to both elderly healthy controls and patients with cognitive impairment without brain amyloidosis, the abundance of *Escherichia*-*Shigella* in the fecal microbiome was higher in elderly people with cognitive impairment and brain amyloidosis [2]. The same study further demonstrated that the abundance of *Escherichia*-*Shigella* was positively correlated with levels of proinflammatory cytokines such as IL-1*β*, CXCL2, and NLRP3. Thus, these findings raise the possibility that bacterial infection might induce A*β* accumulation and promote the chronic inflammatory responses that drive AD pathogenesis [4, 28, 29]. Nevertheless, further investigation is required to determine whether *Escherichia*-*Shigella* is a risk factor for AD and how these bacteria trigger the inflammatory and pathologic processes.

In the present study, Verrucomicrobia increased consistently in APP/PS1 mice relative to age-matched WT mice at various ages, which was reflected by increased abundances of Verrucomicrobiaceae and *Akkermansia* at the family and genus levels, respectively. In addition, there was a trend towards elevated abundances of Verrucomicrobia and *Akkermansia* in older mice irrespective of genotype, suggesting that these bacterial taxa generally expand with age. Intriguingly, these differences were not observed in the 3-month-old groups. Indeed, the ratio of the abundance of Firmicutes to Bacteroidetes increased dramatically from 2 to 3 months and then declined at 6 months in both the WT and APP/PS1 mice, which indicates a special time point when the microbial community changes at the age of 3 months. This phenomenon may explain why few microbial taxa were detected between the 2 groups at this age. The abundance of Verrucomicrobia was recently reported to increase dramatically in older APP/PS1 mice, which suggests a potential association between these bacteria and AD progression [24]. *Akkermansia muciniphila*, the single known species within its genus, is a mucin-degrading bacterium that has attracted considerable attention in recent years because of its immunomodulatory effects on PD-1-based antitumor immunotherapy [61]. Accordingly, decreased *Akkermansia* abundance is associated with increased risks of inflammatory bowel disease, obesity, and type 2 diabetes [62, 63]. Furthermore, patients with advanced dementia and *Clostridium difficile* colonization, colon cancer, or multiple sclerosis exhibit an elevated abundance of *Akkermansia* [64–66]. Together with our current findings, these studies collectively indicate that *Akkermansia* has physiological significance in both healthy and disease conditions. Nevertheless, further studies are necessary to identify how *Akkermansia* modulates the immune system during AD progression.

Furthermore, recent evidence suggests that the metabolic byproducts of microbes, such as short-chain fatty acids, might be involved in the modulation of the peripheral and central immune pathways and might thus influence brain functions [67, 68]. In the present study, APP/PS1 mice exhibited a significant increase in the abundance of *Blautia* at 6 months, when distinctive AD pathologic characteristics such as A*β* deposition are present in the brain. As a genus within family Lachnospiraceae in phylum Firmicutes, *Blautia* degrades polysaccharides into short-chain fatty acids that the host can use as an energy supply [69]. AD patients are reported to have an increased abundance of *Blautia*, which is correlated with more severe AD pathology, namely, lower levels of A*β*_42_/A*β*_40_ and higher levels of *p*-tau and *p*-tau/A*β*_42_ in the cerebrospinal fluid [22]. Another study focusing on microbiota-derived extracellular vesicles in the blood also reports substantial elevation of *Blautia* abundance in APP/PS1 mice [70]. Moreover, an aberrant abundance of *Blautia* has been observed in other CNS disorders that share many characteristics with AD, namely, Parkinson's disease and multiple sclerosis [52, 71]. These findings collectively suggest that *Blautia* might be a microbial indicator of disease state in certain CNS disorders and might thus serve as a potential biomarker for the evaluation of AD therapies.

Besides potential associations with disease states, alterations in the composition of the intestinal microbiome caused by probiotics or beneficial bacteria can confer health benefits to the host [72–74]. AD patients are reported to exhibit a decreased abundance of Actinobacteria, particularly *Bifidobacterium*, which are considered important probiotics associated with the inhibition of pathogenic bacteria and modulation of systemic immune responses [22, 75]. However, in the present study, the abundances of Actinobacteria and *Bifidobacterium* were greater in APP/PS1 mice than their WT controls at 2 months. This inconsistency might reflect the dynamic and complex nature of microbiota composition under different genetic and environmental circumstances; alternatively, it might indicate that gut microbiota undergoes regulation or compensation in response to early-life inflammatory insult. The brain-gut axis allows bidirectional communication between the CNS and the enteric nervous system, linking the emotional and cognitive centers of the brain with peripheral intestinal functions [76]. Thus, microbiological therapies might be able to restore cognitive health and treat neurological diseases via the modulation of gut microbiota communities.

All animals used in this study were male. During the progression of AD, the male and female mice differ in many aspects, such as body fluid biomarkers, hippocampal atrophy, cognitive decline, and other pathological footprints [77]. Furthermore, the pathological progression in female mice might be affected by the hormone cycle [78]. Previous studies also show that there are sex differences in the gut microbiome [79] and that the gut microbiome can be affected by hormones in female mice [80]. Recent reports show that antibiotic manipulation of the gut microbiome alleviates AD-related pathology in male mice but not female mice [27, 37]. Thus, to minimize the variation introduced by sex differences, we only included male animals in this study. In humans, the prevalence of AD or other dementias is 11% in males and 16% in females at the age of 71 and older, respectively [81]. This discrepancy might be due to the longer lifespan of women or related health factors as well as interactions between genetic factors and sex hormones [82–84]. However, the causes of the observed discrepancy remain unclear.

In summary, the present longitudinal study shows that microbiota composition in WT mice and an AD mouse model begins diverging early, prior to amyloid plaque formation and neuroinflammation in the cerebral cortex. Meanwhile, at later ages, the microbial composition in AD mouse models shifts towards an inflammation-related bacterial profile (i.e., increases in the abundance of *Escherichia*-*Shigella* and *Desulfovibrio*), coinciding with abundant microglial accumulation at sites of amyloid deposition in the brain. Nevertheless, the molecular mechanisms and signaling cascades that link the dysregulation of gut microbiota and pathogenesis of AD await further investigation.

## Figures and Tables

**Table 1:** Number of mouse fecal samples collected in each age group.

Mouse group	1 month (*n*)	2 months (*n*)	3 months (*n*)	6 months (*n*)	9 months (*n*)
WT mice	14	17	17	31	18
APP/PS1 mice	21	24	24	34	18

WT: wild type.

**Table 2:** Ratio of the abundance of Firmicutes to Bacteroidetes.

Mouse group	1 month	2 months	3 months	6 months	9 months
WT mice	0.35	0.40	1.13	0.62	0.93
APP/PS1 mice	0.38	0.39	1.30	0.85	1.01

WT: wild type.

**Table 3:** Comparison of alpha diversity between the wild-type and APP/PS1 mice.

	ACE (*p* value)	Chao1 (*p* value)	OTU (*p* value)
WT-APP/PS1 1 m	0.479859	0.277845	0.433141
WT-APP/PS1 2 m	0.06619	0.044915^∗^	0.11163
WT-APP/PS1 3 m	0.173755	0.241569	0.280179
WT-APP/PS1 6 m	0.302173	0.282751	0.481211
WT-APP/PS1 9 m	0.008454^∗∗^	0.011844^∗^	0.021394^∗^

OTU: operational taxonomic unit; WT: wild type. Alpha diversity was assessed by the Wilcoxon rank-sum test; ^∗^*p* < 0.05, ^∗∗^*p* < 0.01. WT-APP/PS1 1 m, WT-APP/PS1 2 m, WT-APP/PS1 3 m, WT-APP/PS1 6 m, and WT-APP/PS1 9 m show comparisons between WT mice and APP/PS1 transgenic mice at 1, 2, 3, 6, and 9 months of age, respectively.

**Table 4:** Comparison of beta diversity between the wild-type and APP/PS1 mice by *Pairwise*.*adonis* (*unifrac_1*, *detail*).

	F.Model	*R* ^2^	*p* value	Adjusted *p* value	Significance
WT-APP/PS1 1 m	0.7535174	0.02232412	0.817	0.817	ns
WT-APP/PS1 2 m	1.861077	0.04668909	0.035	0.035	^∗^
WT-APP/PS1 3 m	1.614989	0.03976336	0.052	0.052	ns
WT-APP/PS1 6 m	1.523908	0.03016212	0.045	0.045	^∗^
WT-APP/PS1 9 m	1.171244	0.03330119	0.246	0.246	ns

ns: not significant; WT: wild type. Beta diversity was assessed by PERMANOVA; ^∗^*p* < 0.05. WT-APP/PS1 1 m, WT-APP/PS1 2 m, WT-APP/PS1 3 m, WT-APP/PS1 6 m, and WT-APP/PS1 9 m show comparisons between WT mice and APP/PS1 transgenic mice at 1, 2, 3, 6, and 9 months of age, respectively.

## Data Availability

The data used to support the findings of this study are included within the article, and the Mice Fecal 16S rDNA Amplification Raw Sequence Reads data have been uploaded on the NCBI SRA database: https://www.ncbi.nlm.nih.gov/bioproject/543965 (project ID: 543965).

## Supplementary Materials

Supplementary Materials Table S1. Primer sequences for amplification and sequencing of 16S rRNA genes .Table S2. Significantly different taxa between WT and AD mice at phylum, family and genus level. Figure S1. Length of sequence reads of all samples.Figure S2. (A–E) Heatmaps showing the differentially abundant taxa at the OTU level in wild-type (WT) and APP/PS1 mice at 1, 2, 3, 6, and 9 months of age, respectively. Each column in the heatmap represents a mouse fecal sample, and each row represents an OTU-level taxon that is differentially abundant in WT or APP/PS1 mice. The 16S rRNA sequence-based cluster tree for the differential OTUs is presented on the left side of the heatmap. 1m, 2m, 3m, 6m, 9m indicate mice at 1, 2, 3, 6, and 9 months of age, respectively.Figure S3. Differentially represented bacterial taxa of WT (A) and APP/PS1 (B) mice at different ages, as revealed by LEfSe analysis. 1m, 2m, 3m, 6m, 9m indicate mice at 1, 2, 3, 6, and 9 months of age, respectively.
