# Supplementary material for: Gut Microbiome Alterations Precede Cerebral Amyloidosis and Microglial Pathology in a Mouse Model of Alzheimer's Disease
Source: Biomed Res Int. 2020 May 24;2020:8456596. doi: 10.1155/2020/8456596 (PMC7273394; doi:10.1155/2020/8456596)
Supplement: Supplementary Materials — Table S1. Primer sequences for amplification and sequencing of 16S rRNA genes .Table S2. Significantly different taxa between WT and AD mice at phylum, family and genus level. Figure S1. Length of sequence reads of all samples.Figure S2. (A–E) Heatmaps showing the differentially abundant taxa at the OTU level in wild-type (WT) and APP/PS1 mice at 1, 2, 3, 6, and 9 months of age, respectively. Each column in the heatmap represents a mouse fecal sample, and each row represents an OTU-level taxon that is differentially abundant in WT or APP/PS1 mice. The 16S rRNA sequence-based cluster tree for the differential OTUs is presented on the left side of the heatmap. 1m, 2m, 3m, 6m, 9m indicate mice at 1, 2, 3, 6, and 9 months of age, respectively.Figure S3. Differentially represented bacterial taxa of WT (A) and APP/PS1 (B) mice at different ages, as revealed by LEfSe analysis. 1m, 2m, 3m, 6m, 9m indicate mice at 1, 2, 3, 6, and 9 months of age, respectively. [file 8456596.f1.pdf]

Table S1. Primer sequences for amplification and sequencing of 16S rRNA genes

Forward Primer Linker	Forward Primer	Read 1 Sequencing Primer
GT	GTGCCAGCMGCCGCGGTAA	TATGGTAATTGTGTGCCAGCMGCCGCGGTAA

Golay barcode	Reverse Primer Pad	Reverse Primer Linker	Reverse Primer	Primer For PCR
TCCCTTGTCTCC	AGTCAGTCAG	CC	GGACTACHVGGGTWTCTAAT	TCCCTTGTCTCCAGTCAGTCAGCCGGACTACHVGGGTWTCTAAT
ACGAGACTGATT	AGTCAGTCAG	CC	GGACTACHVGGGTWTCTAAT	ACGAGACTGATTAGTCAGTCAGCCGGACTACHVGGGTWTCTAAT
GCTGTACGGATT	AGTCAGTCAG	CC	GGACTACHVGGGTWTCTAAT	GCTGTACGGATTAGTCAGTCAGCCGGACTACHVGGGTWTCTAAT
ATCACCAGGTGT	AGTCAGTCAG	CC	GGACTACHVGGGTWTCTAAT	ATCACCAGGTGTAGTCAGTCAGCCGGACTACHVGGGTWTCTAAT
TGGTCAACGATA	AGTCAGTCAG	CC	GGACTACHVGGGTWTCTAAT	TGGTCAACGATAAGTCAGTCAGCCGGACTACHVGGGTWTCTAAT
ATCGCACAGTAA	AGTCAGTCAG	CC	GGACTACHVGGGTWTCTAAT	ATCGCACAGTAAAGTCAGTCAGCCGGACTACHVGGGTWTCTAAT
GTCGTGTAGCCT	AGTCAGTCAG	CC	GGACTACHVGGGTWTCTAAT	GTCGTGTAGCCTAGTCAGTCAGCCGGACTACHVGGGTWTCTAAT
AGCGGAGGTTAG	AGTCAGTCAG	CC	GGACTACHVGGGTWTCTAAT	AGCGGAGGTTAGAGTCAGTCAGCCGGACTACHVGGGTWTCTAAT
ATCCTTTGGTTC	AGTCAGTCAG	CC	GGACTACHVGGGTWTCTAAT	ATCCTTTGGTTCAGTCAGTCAGCCGGACTACHVGGGTWTCTAAT
TACAGCGCATAC	AGTCAGTCAG	CC	GGACTACHVGGGTWTCTAAT	TACAGCGCATACAGTCAGTCAGCCGGACTACHVGGGTWTCTAAT
ACCGGTATGTAC	AGTCAGTCAG	CC	GGACTACHVGGGTWTCTAAT	ACCGGTATGTACAGTCAGTCAGCCGGACTACHVGGGTWTCTAAT
AATTGTGTCGGA	AGTCAGTCAG	CC	GGACTACHVGGGTWTCTAAT	AATTGTGTCGGAAGTCAGTCAGCCGGACTACHVGGGTWTCTAAT
TGCATACACTGG	AGTCAGTCAG	CC	GGACTACHVGGGTWTCTAAT	TGCATACACTGGAGTCAGTCAGCCGGACTACHVGGGTWTCTAAT
AGTCGAACGAGG	AGTCAGTCAG	CC	GGACTACHVGGGTWTCTAAT	AGTCGAACGAGGAGTCAGTCAGCCGGACTACHVGGGTWTCTAAT
ACCAGTGACTCA	AGTCAGTCAG	CC	GGACTACHVGGGTWTCTAAT	ACCAGTGACTCAAGTCAGTCAGCCGGACTACHVGGGTWTCTAAT
GAATACCAAGTC	AGTCAGTCAG	CC	GGACTACHVGGGTWTCTAAT	GAATACCAAGTCAGTCAGTCAGCCGGACTACHVGGGTWTCTAAT
GTAGATCGTGTA	AGTCAGTCAG	CC	GGACTACHVGGGTWTCTAAT	GTAGATCGTGTAAGTCAGTCAGCCGGACTACHVGGGTWTCTAAT
TAACGTGTGTGC	AGTCAGTCAG	CC	GGACTACHVGGGTWTCTAAT	TAACGTGTGTGCAGTCAGTCAGCCGGACTACHVGGGTWTCTAAT
CATTATGGCGTG	AGTCAGTCAG	CC	GGACTACHVGGGTWTCTAAT	CATTATGGCGTGAGTCAGTCAGCCGGACTACHVGGGTWTCTAAT
CCAATACGCCTG	AGTCAGTCAG	CC	GGACTACHVGGGTWTCTAAT	CCAATACGCCTGAGTCAGTCAGCCGGACTACHVGGGTWTCTAAT
GATCTGCGATCC	AGTCAGTCAG	CC	GGACTACHVGGGTWTCTAAT	GATCTGCGATCCAGTCAGTCAGCCGGACTACHVGGGTWTCTAAT
CAGCTCATCAGC	AGTCAGTCAG	CC	GGACTACHVGGGTWTCTAAT	CAGCTCATCAGCAGTCAGTCAGCCGGACTACHVGGGTWTCTAAT
CAAACAACAGCT	AGTCAGTCAG	CC	GGACTACHVGGGTWTCTAAT	CAAACAACAGCTAGTCAGTCAGCCGGACTACHVGGGTWTCTAAT
GCAACACCATCC	AGTCAGTCAG	CC	GGACTACHVGGGTWTCTAAT	GCAACACCATCCAGTCAGTCAGCCGGACTACHVGGGTWTCTAAT
GCGATATATCGC	AGTCAGTCAG	CC	GGACTACHVGGGTWTCTAAT	GCGATATATCGCAGTCAGTCAGCCGGACTACHVGGGTWTCTAAT
CGAGCAATCCTA	AGTCAGTCAG	CC	GGACTACHVGGGTWTCTAAT	CGAGCAATCCTAAGTCAGTCAGCCGGACTACHVGGGTWTCTAAT
AGTCGTGCACAT	AGTCAGTCAG	CC	GGACTACHVGGGTWTCTAAT	AGTCGTGCACATAGTCAGTCAGCCGGACTACHVGGGTWTCTAAT
GTATCTGCGCGT	AGTCAGTCAG	CC	GGACTACHVGGGTWTCTAAT	GTATCTGCGCGTAGTCAGTCAGCCGGACTACHVGGGTWTCTAAT
CGAGGGAAAGTC	AGTCAGTCAG	CC	GGACTACHVGGGTWTCTAAT	CGAGGGAAAGTCAGTCAGTCAGCCGGACTACHVGGGTWTCTAAT
CAAATTCGGGAT	AGTCAGTCAG	CC	GGACTACHVGGGTWTCTAAT	CAAATTCGGGATAGTCAGTCAGCCGGACTACHVGGGTWTCTAAT
AGATTGACCAAC	AGTCAGTCAG	CC	GGACTACHVGGGTWTCTAAT	AGATTGACCAACAGTCAGTCAGCCGGACTACHVGGGTWTCTAAT
AGTTACGAGCTA	AGTCAGTCAG	CC	GGACTACHVGGGTWTCTAAT	AGTTACGAGCTAAGTCAGTCAGCCGGACTACHVGGGTWTCTAAT
GCATATGCACTG	AGTCAGTCAG	CC	GGACTACHVGGGTWTCTAAT	GCATATGCACTGAGTCAGTCAGCCGGACTACHVGGGTWTCTAAT
CAACTCCCGTGA	AGTCAGTCAG	CC	GGACTACHVGGGTWTCTAAT	CAACTCCCGTGAAGTCAGTCAGCCGGACTACHVGGGTWTCTAAT
TTGCGTTAGCAG	AGTCAGTCAG	CC	GGACTACHVGGGTWTCTAAT	TTGCGTTAGCAGAGTCAGTCAGCCGGACTACHVGGGTWTCTAAT
TACGAGCCCTAA	AGTCAGTCAG	CC	GGACTACHVGGGTWTCTAAT	TACGAGCCCTAAAGTCAGTCAGCCGGACTACHVGGGTWTCTAAT
CACTACGCTAGA	AGTCAGTCAG	CC	GGACTACHVGGGTWTCTAAT	CACTACGCTAGAAGTCAGTCAGCCGGACTACHVGGGTWTCTAAT
TGCAGTCCTCGA	AGTCAGTCAG	CC	GGACTACHVGGGTWTCTAAT	TGCAGTCCTCGAAGTCAGTCAGCCGGACTACHVGGGTWTCTAAT
ACCATAGCTCCG	AGTCAGTCAG	CC	GGACTACHVGGGTWTCTAAT	ACCATAGCTCCGAGTCAGTCAGCCGGACTACHVGGGTWTCTAAT

TCGACATCTCTT	AGTCAGTCAG	CC	GGACTACHVGGGTWTCTAAT	TCGACATCTCTTAGTCAGTCAGCCGGACTACHVGGGTWTCTAAT
GAACACTTTGGA	AGTCAGTCAG	CC	GGACTACHVGGGTWTCTAAT	GAACACTTTGGAAGTCAGTCAGCCGGACTACHVGGGTWTCTAAT
GAGCCATCTGTA	AGTCAGTCAG	CC	GGACTACHVGGGTWTCTAAT	GAGCCATCTGTAAGTCAGTCAGCCGGACTACHVGGGTWTCTAAT
TTGGGTACACGT	AGTCAGTCAG	CC	GGACTACHVGGGTWTCTAAT	TTGGGTACACGTAGTCAGTCAGCCGGACTACHVGGGTWTCTAAT
AAGGCGCTCCTT	AGTCAGTCAG	CC	GGACTACHVGGGTWTCTAAT	AAGGCGCTCCTTAGTCAGTCAGCCGGACTACHVGGGTWTCTAAT
TAATACGGATCG	AGTCAGTCAG	CC	GGACTACHVGGGTWTCTAAT	TAATACGGATCGAGTCAGTCAGCCGGACTACHVGGGTWTCTAAT
TCGGAATTAGAC	AGTCAGTCAG	CC	GGACTACHVGGGTWTCTAAT	TCGGAATTAGACAGTCAGTCAGCCGGACTACHVGGGTWTCTAAT
TGTGAATTCGGA	AGTCAGTCAG	CC	GGACTACHVGGGTWTCTAAT	TGTGAATTCGGAAGTCAGTCAGCCGGACTACHVGGGTWTCTAAT
CATTCGTGGCGT	AGTCAGTCAG	CC	GGACTACHVGGGTWTCTAAT	CATTCGTGGCGTAGTCAGTCAGCCGGACTACHVGGGTWTCTAAT
TACTACGTGGCC	AGTCAGTCAG	CC	GGACTACHVGGGTWTCTAAT	TACTACGTGGCCAGTCAGTCAGCCGGACTACHVGGGTWTCTAAT
GGCCAGTTCCTA	AGTCAGTCAG	CC	GGACTACHVGGGTWTCTAAT	GGCCAGTTCCTAAGTCAGTCAGCCGGACTACHVGGGTWTCTAAT
GATGTTCGCTAG	AGTCAGTCAG	CC	GGACTACHVGGGTWTCTAAT	GATGTTCGCTAGAGTCAGTCAGCCGGACTACHVGGGTWTCTAAT
CTATCTCCTGTC	AGTCAGTCAG	CC	GGACTACHVGGGTWTCTAAT	CTATCTCCTGTCAGTCAGTCAGCCGGACTACHVGGGTWTCTAAT
ACTCACAGGAAT	AGTCAGTCAG	CC	GGACTACHVGGGTWTCTAAT	ACTCACAGGAATAGTCAGTCAGCCGGACTACHVGGGTWTCTAAT
ATGATGAGCCTC	AGTCAGTCAG	CC	GGACTACHVGGGTWTCTAAT	ATGATGAGCCTCAGTCAGTCAGCCGGACTACHVGGGTWTCTAAT
GTCGACAGAGGA	AGTCAGTCAG	CC	GGACTACHVGGGTWTCTAAT	GTCGACAGAGGAAGTCAGTCAGCCGGACTACHVGGGTWTCTAAT
TGTCGCAAATAG	AGTCAGTCAG	CC	GGACTACHVGGGTWTCTAAT	TGTCGCAAATAGAGTCAGTCAGCCGGACTACHVGGGTWTCTAAT
CATCCCTCTACT	AGTCAGTCAG	CC	GGACTACHVGGGTWTCTAAT	CATCCCTCTACTAGTCAGTCAGCCGGACTACHVGGGTWTCTAAT
TATACCGCTGCG	AGTCAGTCAG	CC	GGACTACHVGGGTWTCTAAT	TATACCGCTGCGAGTCAGTCAGCCGGACTACHVGGGTWTCTAAT
AGTTGAGGCATT	AGTCAGTCAG	CC	GGACTACHVGGGTWTCTAAT	AGTTGAGGCATTAGTCAGTCAGCCGGACTACHVGGGTWTCTAAT
ACAATAGACACC	AGTCAGTCAG	CC	GGACTACHVGGGTWTCTAAT	ACAATAGACACCAGTCAGTCAGCCGGACTACHVGGGTWTCTAAT
CGGTCAATTGAC	AGTCAGTCAG	CC	GGACTACHVGGGTWTCTAAT	CGGTCAATTGACAGTCAGTCAGCCGGACTACHVGGGTWTCTAAT
GTGGAGTCTCAT	AGTCAGTCAG	CC	GGACTACHVGGGTWTCTAAT	GTGGAGTCTCATAGTCAGTCAGCCGGACTACHVGGGTWTCTAAT
GCTCGAAGATTC	AGTCAGTCAG	CC	GGACTACHVGGGTWTCTAAT	GCTCGAAGATTCAGTCAGTCAGCCGGACTACHVGGGTWTCTAAT
AGGCTTACGTGT	AGTCAGTCAG	CC	GGACTACHVGGGTWTCTAAT	AGGCTTACGTGTAGTCAGTCAGCCGGACTACHVGGGTWTCTAAT
TCTCTACCACTC	AGTCAGTCAG	CC	GGACTACHVGGGTWTCTAAT	TCTCTACCACTCAGTCAGTCAGCCGGACTACHVGGGTWTCTAAT
ACTTCCAACTTC	AGTCAGTCAG	CC	GGACTACHVGGGTWTCTAAT	ACTTCCAACTTCAGTCAGTCAGCCGGACTACHVGGGTWTCTAAT
CTCACCTAGGAA	AGTCAGTCAG	CC	GGACTACHVGGGTWTCTAAT	CTCACCTAGGAAAGTCAGTCAGCCGGACTACHVGGGTWTCTAAT
GTGTTGTCGTGC	AGTCAGTCAG	CC	GGACTACHVGGGTWTCTAAT	GTGTTGTCGTGCAGTCAGTCAGCCGGACTACHVGGGTWTCTAAT
CCACAGATCGAT	AGTCAGTCAG	CC	GGACTACHVGGGTWTCTAAT	CCACAGATCGATAGTCAGTCAGCCGGACTACHVGGGTWTCTAAT
TATCGACACAAG	AGTCAGTCAG	CC	GGACTACHVGGGTWTCTAAT	TATCGACACAAGAGTCAGTCAGCCGGACTACHVGGGTWTCTAAT
GATTCCGGCTCA	AGTCAGTCAG	CC	GGACTACHVGGGTWTCTAAT	GATTCCGGCTCAAGTCAGTCAGCCGGACTACHVGGGTWTCTAAT
CGTAATTGCCGC	AGTCAGTCAG	CC	GGACTACHVGGGTWTCTAAT	CGTAATTGCCGCAGTCAGTCAGCCGGACTACHVGGGTWTCTAAT
GGTGACTAGTTC	AGTCAGTCAG	CC	GGACTACHVGGGTWTCTAAT	GGTGACTAGTTCAGTCAGTCAGCCGGACTACHVGGGTWTCTAAT
ATGGGTTCCGTC	AGTCAGTCAG	CC	GGACTACHVGGGTWTCTAAT	ATGGGTTCCGTCAGTCAGTCAGCCGGACTACHVGGGTWTCTAAT
TAGGCATGCTTG	AGTCAGTCAG	CC	GGACTACHVGGGTWTCTAAT	TAGGCATGCTTGAGTCAGTCAGCCGGACTACHVGGGTWTCTAAT
AACTAGTTCAGG	AGTCAGTCAG	CC	GGACTACHVGGGTWTCTAAT	AACTAGTTCAGGAGTCAGTCAGCCGGACTACHVGGGTWTCTAAT
ATTCTGCCGAAG	AGTCAGTCAG	CC	GGACTACHVGGGTWTCTAAT	ATTCTGCCGAAGAGTCAGTCAGCCGGACTACHVGGGTWTCTAAT
AGCATGTCCCGT	AGTCAGTCAG	CC	GGACTACHVGGGTWTCTAAT	AGCATGTCCCGTAGTCAGTCAGCCGGACTACHVGGGTWTCTAAT
GTACGATATGAC	AGTCAGTCAG	CC	GGACTACHVGGGTWTCTAAT	GTACGATATGACAGTCAGTCAGCCGGACTACHVGGGTWTCTAAT
GTGGTGGTTTCC	AGTCAGTCAG	CC	GGACTACHVGGGTWTCTAAT	GTGGTGGTTTCCAGTCAGTCAGCCGGACTACHVGGGTWTCTAAT
TAGTATGCGCAA	AGTCAGTCAG	CC	GGACTACHVGGGTWTCTAAT	TAGTATGCGCAAAGTCAGTCAGCCGGACTACHVGGGTWTCTAAT
TGCGCTGAATGT	AGTCAGTCAG	CC	GGACTACHVGGGTWTCTAAT	TGCGCTGAATGTAGTCAGTCAGCCGGACTACHVGGGTWTCTAAT
ATGGCTGTCAGT	AGTCAGTCAG	CC	GGACTACHVGGGTWTCTAAT	ATGGCTGTCAGTAGTCAGTCAGCCGGACTACHVGGGTWTCTAAT

GTTCTCTTCTCG	AGTCAGTCAG	CC	GGACTACHVGGGTWTCTAAT	GTTCTCTTCTCGAGTCAGTCAGCCGGACTACHVGGGTWTCTAAT
CGTAAGATGCCT	AGTCAGTCAG	CC	GGACTACHVGGGTWTCTAAT	CGTAAGATGCCTAGTCAGTCAGCCGGACTACHVGGGTWTCTAAT
GCGTTCTAGCTG	AGTCAGTCAG	CC	GGACTACHVGGGTWTCTAAT	GCGTTCTAGCTGAGTCAGTCAGCCGGACTACHVGGGTWTCTAAT
GTTGTTCTGGGA	AGTCAGTCAG	CC	GGACTACHVGGGTWTCTAAT	GTTGTTCTGGGAAGTCAGTCAGCCGGACTACHVGGGTWTCTAAT
GGACTTCCAGCT	AGTCAGTCAG	CC	GGACTACHVGGGTWTCTAAT	GGACTTCCAGCTAGTCAGTCAGCCGGACTACHVGGGTWTCTAAT
CTCACAACCGTG	AGTCAGTCAG	CC	GGACTACHVGGGTWTCTAAT	CTCACAACCGTGAGTCAGTCAGCCGGACTACHVGGGTWTCTAAT
CTGCTATTCCTC	AGTCAGTCAG	CC	GGACTACHVGGGTWTCTAAT	CTGCTATTCCTCAGTCAGTCAGCCGGACTACHVGGGTWTCTAAT
ATGTCACCGCTG	AGTCAGTCAG	CC	GGACTACHVGGGTWTCTAAT	ATGTCACCGCTGAGTCAGTCAGCCGGACTACHVGGGTWTCTAAT
TGTAACGCCGAT	AGTCAGTCAG	CC	GGACTACHVGGGTWTCTAAT	TGTAACGCCGATAGTCAGTCAGCCGGACTACHVGGGTWTCTAAT
AGCAGAACATCT	AGTCAGTCAG	CC	GGACTACHVGGGTWTCTAAT	AGCAGAACATCTAGTCAGTCAGCCGGACTACHVGGGTWTCTAAT
TGGAGTAGGTGG	AGTCAGTCAG	CC	GGACTACHVGGGTWTCTAAT	TGGAGTAGGTGGAGTCAGTCAGCCGGACTACHVGGGTWTCTAAT
TTGGCTCTATTC	AGTCAGTCAG	CC	GGACTACHVGGGTWTCTAAT	TTGGCTCTATTCAGTCAGTCAGCCGGACTACHVGGGTWTCTAAT
GATCCCACGTAC	AGTCAGTCAG	CC	GGACTACHVGGGTWTCTAAT	GATCCCACGTACAGTCAGTCAGCCGGACTACHVGGGTWTCTAAT
TACCGCTTCTTC	AGTCAGTCAG	CC	GGACTACHVGGGTWTCTAAT	TACCGCTTCTTCAGTCAGTCAGCCGGACTACHVGGGTWTCTAAT
TGTGCGATAACA	AGTCAGTCAG	CC	GGACTACHVGGGTWTCTAAT	TGTGCGATAACAAGTCAGTCAGCCGGACTACHVGGGTWTCTAAT
GATTATCGACGA	AGTCAGTCAG	CC	GGACTACHVGGGTWTCTAAT	GATTATCGACGAAGTCAGTCAGCCGGACTACHVGGGTWTCTAAT
GCCTAGCCCAAT	AGTCAGTCAG	CC	GGACTACHVGGGTWTCTAAT	GCCTAGCCCAATAGTCAGTCAGCCGGACTACHVGGGTWTCTAAT
GATGTATGTGGT	AGTCAGTCAG	CC	GGACTACHVGGGTWTCTAAT	GATGTATGTGGTAGTCAGTCAGCCGGACTACHVGGGTWTCTAAT

Table S2. Significantly different taxa between WT and AD mice at phylum, family and genus level.

Taxon	Occupancy (%; Mean±SD) WT	Occupancy (%; Mean±SD) APP/PS1		Fold change
**1-month-old**				
f__Enterobacteriaceae	0.01 ±0.01	0.80 ±3.57	↑	132.9
**2-month-old**				
p__Verrucomicrobia	0.02 ±0.04	0.26 ±0.66	↑	15.1
f__Verrucomicrobiaceae	0.02 ±0.04	0.26 ±0.66	↑	15.1
g__Akkermansia	0.02 ±0.04	0.26 ±0.66	↑	15.1
f__Prevotellaceae	0.01 ±0.02	0.71 ±1.79	↑	56.0
g__Prevotellaceae_UCG-001	0.01 ±0.02	0.68 ±1.74	↑	55.9
g__Alloprevotella	0.000 ±0.001	0.025 ±0.050	↑	52.0
p__Actinobacteria	0.50 ±0.43	1.37 ±1.26	↑	2.7
f__Bifidobacteriaceae	0.44 ±0.42	1.24 ±1.29	↑	2.8
g__Bifidobacterium	0.44 ±0.42	1.24 ±1.29	↑	2.8
f__Erysipelotrichaceae	0.03 ±0.03	0.07 ±0.09	↑	2.5
g__Allobaculum	0.001 ±0.002	0.039 ±0.097	↑	39.4
g__Erysipelatoclostridium	0.007 ±0.010	0.010 ±0.007	↑	1.6
**3-month-old**				
g__Lachnospiraceae_UCG-001	1.38 ±1.91	4.07 ±5.24	↑	3.0
g__Ruminiclostridium_5	0.15 ±0.11	0.08 ±0.05	↓	-1.7
g__Ruminococcaceae_UCG-010	0.02 ±0.01	0.01 ±0.01	↓	-1.7
**6-month-old**				
p__Verrucomicrobia	0.08 ±0.23	0.35 ±0.86	↑	4.4
f__Verrucomicrobiaceae	0.08 ±0.23	0.35 ±0.86	↑	4.4
g__Akkermansia	0.08 ±0.23	0.35 ±0.86	↑	4.4
f__Bacteroidaceae	1.18 ±1.57	0.36 ±0.30	↓	-3.3
g__Bacteroides	1.18 ±1.57	0.36 ±0.30	↓	-3.3
f__Rikenellaceae	3.15 ±2.36	1.88 ±1.62	↓	-1.7
g__Rikenellaceae_RC9_gut_group	2.65 ±2.26	1.47 ±1.32	↓	-1.8
f__Enterobacteriaceae	0.008 ±0.012	0.049 ±0.152	↑	6.0
g__Escherichia_Shigella	0.008 ±0.012	0.049 ±0.152	↑	6.0
g__Blautia	0.08 ±0.07	0.16 ±0.16	↑	2.0
g__Tyzzerella	0.008 ±0.006	0.014 ±0.010	↑	1.8
g__Family_XIII_UCG-001	0.01 ±0.01	0.02 ±0.02	↑	1.6
g__Turicibacter	0.001 ±0.002	0.052 ±0.099	↑	74.6
g__Unclassified	1.25 ±1.11	2.19 ±1.64	↑	1.8
**9-month-old**				
p__Verrucomicrobia	0.24 ±0.93	1.46 ±5.58	↑	6.1
f__Verrucomicrobiaceae	0.24 ±0.93	1.46 ±5.58	↑	6.1
g__Akkermansia	0.24 ±0.93	1.46 ±5.58	↑	6.1
p__Proteobacteria	0.94 ±0.65	1.70 ±1.09	↑	1.8

f__Desulfovibrionaceae	0.59 ±0.60	1.06 ±0.77	↑	1.8
g__Desulfovibrio	0.59 ±0.60	1.06 ±0.77	↑	1.8
f__Clostridiales_vadinBB60_group	0.44 ±0.31	1.15 ±0.99	↑	2.6
g__uncultured_bacterium_f_Clostridiales_vadinBB60_group	0.42 ±0.30	1.14 ±0.98	↑	2.7
g__Lachnospiraceae_UCG-001	0.98 ±1.90	1.96 ±3.13	↑	2.0
g__Lachnospiraceae_UCG-006	0.105 ±0.073	0.067 ±0.068	↓	-1.6
g__Ruminococcus_1	0.015 ±0.026	0.058 ±0.075	↑	3.8
g__[Eubacterium]_nodatum_group	0.011 ±0.008	0.006 ±0.004	↓	-2.0
f__Carnobacteriaceae	0.028 ±0.067	0.005 ±0.013	↓	-5.7
g__Atopostipes	0.028 ±0.067	0.005 ±0.013	↓	-5.7
g__Alistipes	0.41 ±0.35	0.26 ±0.32	↓	-1.6
f__Unclassified	0.25 ±0.38	0.78 ±0.88	↑	3.1
g__Unclassified	1.08 ±1.01	2.22 ±1.45	↑	2.0

The percent compositions of microbiomes at the level of phylum, family and genus, for which occupancy differed in APP/PS1 mice as compared to WT controls. Microbiomes with occupancies higher than 0.01% in either WT or APP/PS1 mice were shown. The increase and decrease of the mean occupancy in APP/PS1 mice are indicated by ↑ and ↓ , respectively.

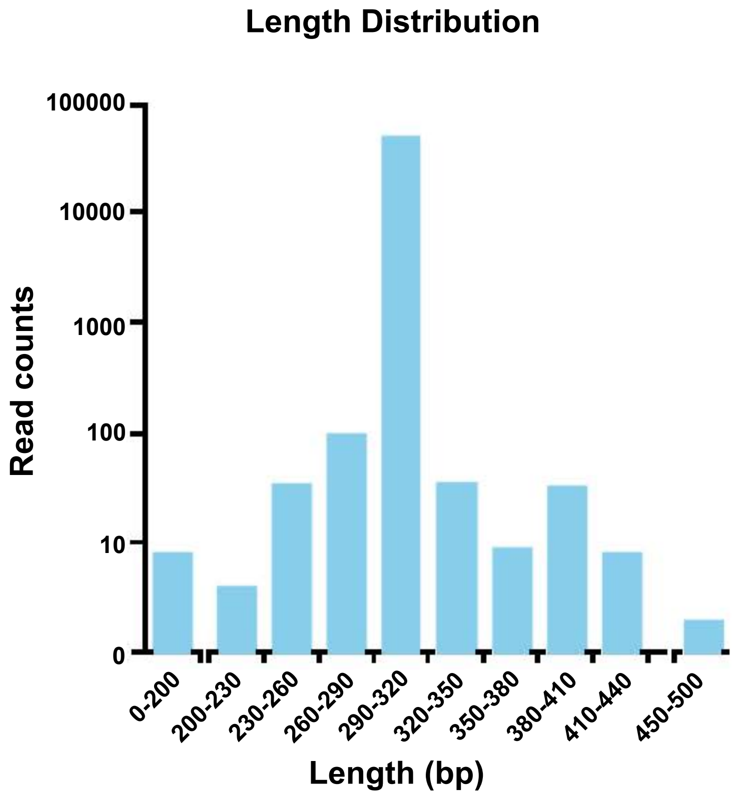

Figure S1. Length of sequence reads of all samples.

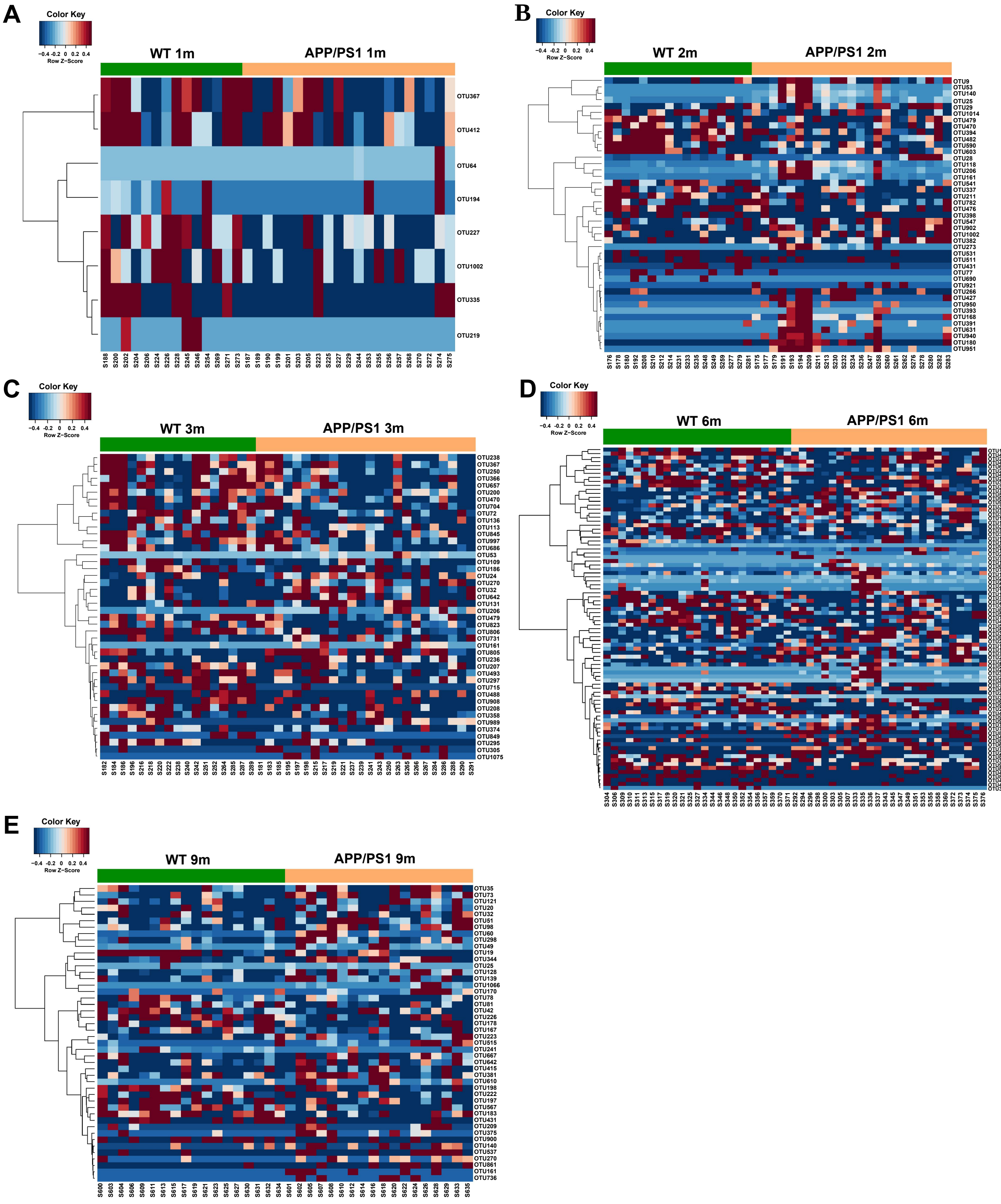

Figure S2. (A–E) Heatmaps showing the differentially abundant taxa at the OTU level in wild-type (WT) and APP/PS1 mice at 1, 2, 3, 6, and 9 months of age, respectively. Each column in the heatmap represents a mouse fecal sample, and each row represents an OTU-level taxon that is differentially abundant in WT or APP/PS1 mice. The 16S rRNA sequence-based cluster tree for the differential OTUs is presented on the left side of the heatmap. 1m, 2m, 3m, 6m, 9m indicate mice at 1, 2, 3, 6, and 9 months of age, respectively.

A

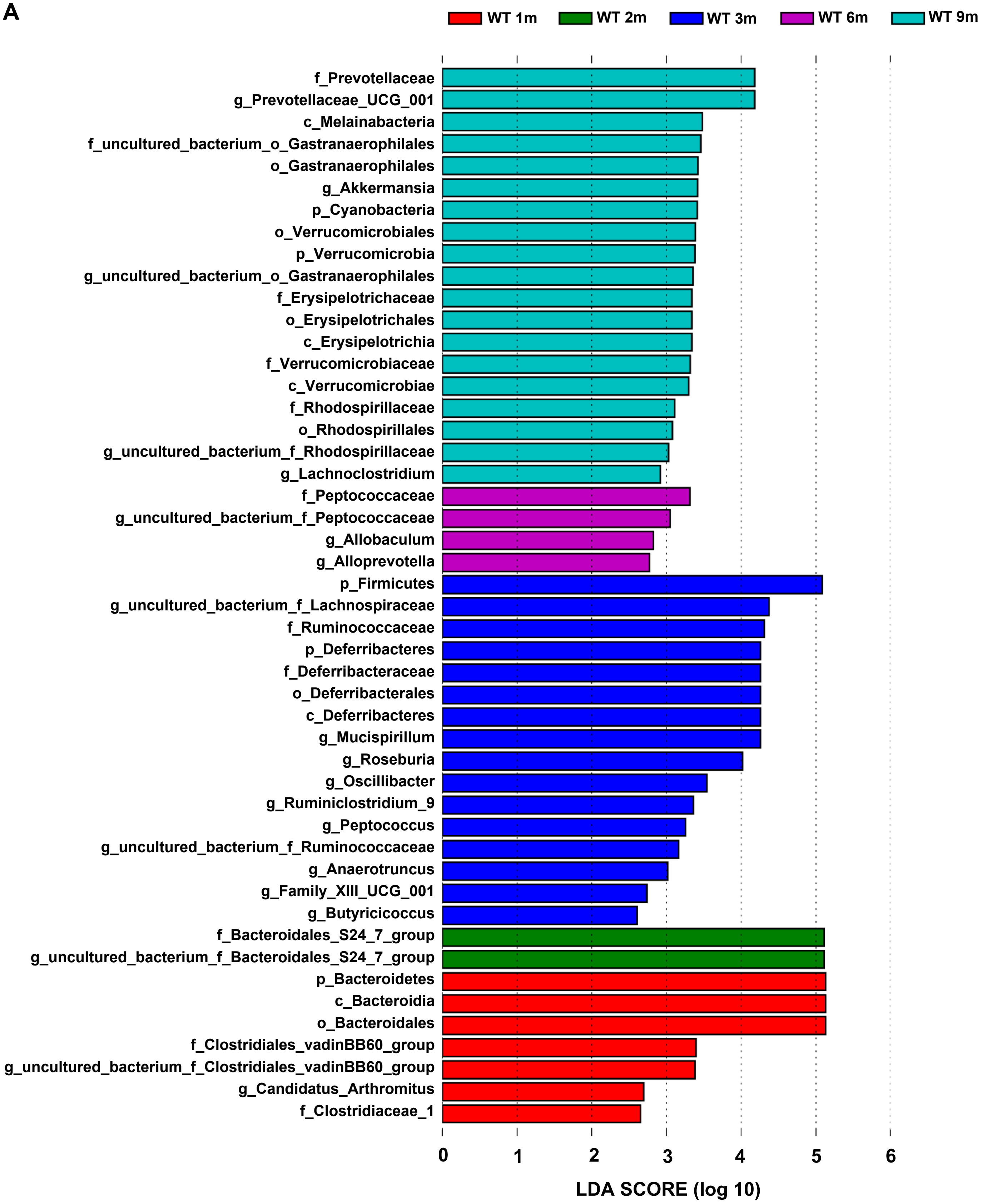
WT 1m
WT 2m
WT 3m
WT 6m
WT 9m
f_Prevotellaceae
g_Prevotellaceae_UCG_001
c_Melainabacteria
f_uncultured_bacterium_o_Gastranaerophilales
o_Gastranaerophilales
g_Akkermansia
p_Cyanobacteria
o_Verrucomicrobiales
p_Verrucomicrobia
g_uncultured_bacterium_o_Gastranaerophilales
f_Erysipelotrichaceae
o_Erysipelotrichales
c_Erysipelotrichia
f_Verrucomicrobiaceae
c_Verrucomicrobiae
f_Rhodospirillaceae
o_Rhodospirillales
g_uncultured_bacterium_f_Rhodospirillaceae
g_Lachnoclostridium
f_Peptococcaceae
g_uncultured_bacterium_f_Peptococcaceae
g_Allobaculum
g_Alloprevotella
p_Firmicutes
g_uncultured_bacterium_f_Lachnospiraceae
f_Ruminococcaceae
p_Deferribacteres
f_Deferribacteraceae
o_Deferribacterales
c_Deferribacteres
g_Mucispirillum
g_Roseburia
g_Oscillibacter
g_Ruminiclostridium_9
g_Peptococcus
g_uncultured_bacterium_f_Ruminococcaceae
g_Anaerotruncus
g_Family_XIII_UCG_001
g_Butyricicoccus
f_Bacteroidales_S24_7_group
g_uncultured_bacterium_f_Bacteroidales_S24_7_group
p_Bacteroidetes
c_Bacteroidia
o_Bacteroidales
f_Clostridiales_vadinBB60_group
g_uncultured_bacterium_f_Clostridiales_vadinBB60_group
g_Candidatus_Arthromitus
f_Clostridiaceae_1
0
1
2
3
4
5
6
LDA SCORE (log 10)

B

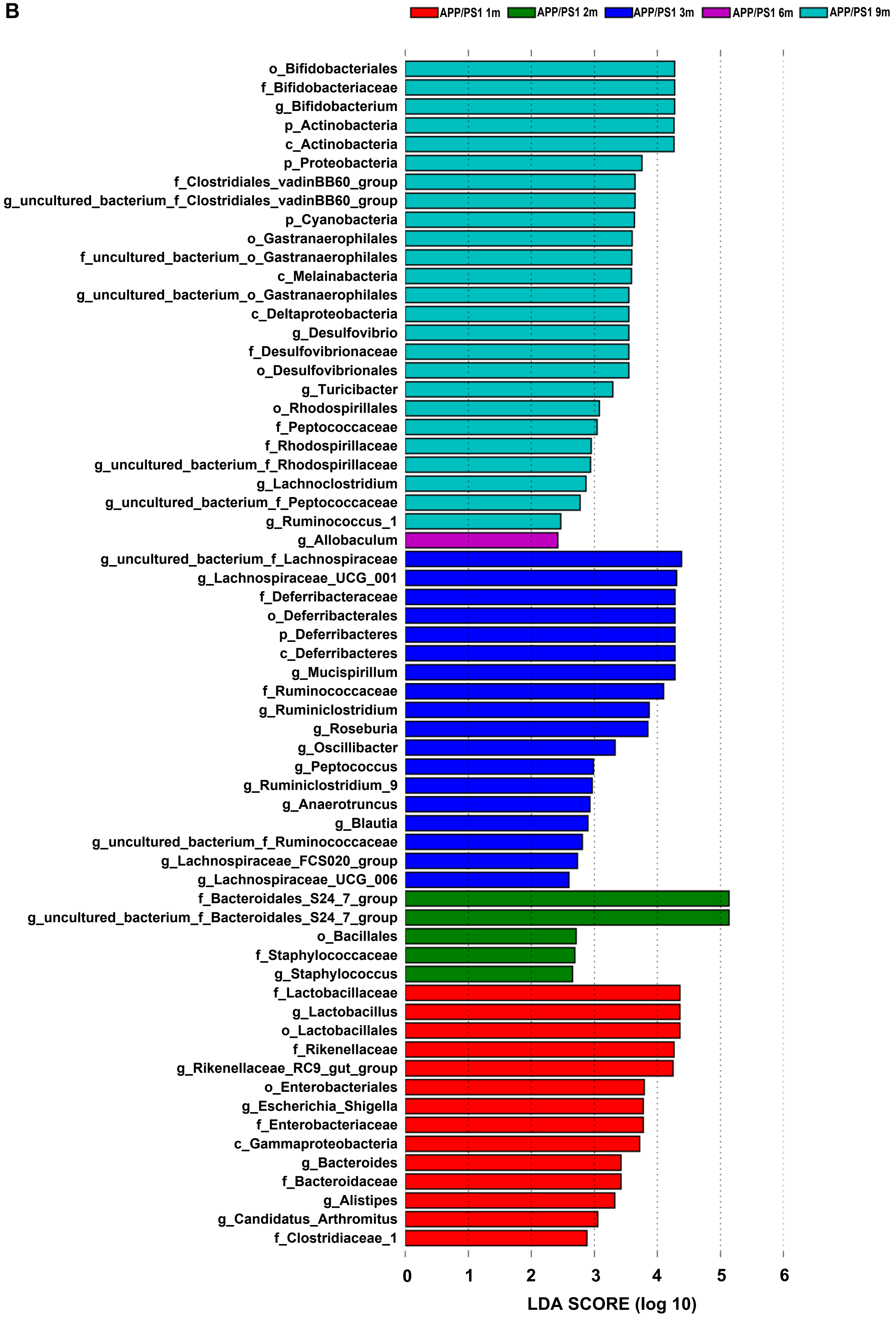

Figure S3. Differentially represented bacterial taxa of WT **(A)** and APP/PS1 **(B)** mice at different ages, as revealed by LEfSe analysis. 1m, 2m, 3m, 6m, 9m indicate mice at 1, 2, 3, 6, and 9 months of age, respectively.
